# Case report of Tourniquet ALPPS and simultaneous sleeve gastrectomy: A valuable association to achieve an adequate future liver remnant in obese patients

**DOI:** 10.1097/MD.0000000000020748

**Published:** 2020-08-21

**Authors:** Roberto Brusadin, Víctor López-López, David Ruiz de Angulo, Asunción López-Conesa, Álvaro Navarro-Barrios, Albert Caballero-Planes, Pascual Parrilla-Paricio, Ricardo Robles-Campos

**Affiliations:** a Department of General, Visceral and Transplantation Surgery; b Department of Pathology, Virgen de la Arrixaca University Hospital, IMIB-Arrixaca, Murcia, Spain.

**Keywords:** ALPPS, colorectal liver metastases, sleeve gastrectomy

## Abstract

**Introduction:**

Obesity represents a risk factor in case of major hepatectomy, because the future liver remnant (FLR) must be proportional with body weight. To avoid post-hepatectomy liver failure, and further increase the ratio between FLR and body weight, we performed a bariatric procedure in the first stage of the ALPPS technique.

**Patient concerns:**

Fifty-four-year-old woman, with morbid obesity (BMI 58.5) and type II diabetes mellitus, was scheduled for a major hepatectomy due to multiple colorectal liver metastases

**Diagnosis:**

Six months before, the patient was diagnosed with colorectal cancer and synchronous liver metastases. She was initially treated with sigmoidectomy and chemotherapy. After partial response of the liver metastases, we considered a liver resection but the FLR was very low, especially in relation to her BMI.

**Intervention:**

We planned a novel approach and, for the first time, we performed a sleeve gastrectomy during the first stage of Tourniquet ALPPS (T-ALPPS). After achieving an adequate FLR, we successfully completed the major hepatectomy during the second stage of T-ALPPS.

**Outcome:**

The association between sleeve gastrectomy and T-ALPPS produced an increase of FLR/body weight ratio up to 0.8 that allowed completing a right trisectionectomy in the second stage of ALPPS. The major hepatectomy was performed without severe complications, and several months after surgery the patient is still alive without any recurrence Conclusion: Despite obesity represents a risk factor involved in the carcinogenesis, the role of the bariatric surgery in the oncological setting is not well established. In this clinical case, we benefited from the weight loss produced by bariatric surgery combined with an effective hypertrophy technique and chemotherapy. These findings suggest that bariatric surgery could be useful for obese patients with liver malignancy and need for extended hepatectomy.

## 1 Introduction

Preserving an adequate future liver remnant (FLR) after major hepatectomy is essential to prevent post-hepatectomy liver failure (PHLF). Several factors such as volume, quality, and function of the liver must be considered to establish the limit for a safe resection. Obesity represents an important risk factor for developing post-operative complications and may even be a contraindication for surgery. FLR should be higher than 25% in patients with healthy liver and above 35% in patients with impaired liver function or after chemotherapy.^[1,2]^ Recently, extrapolating the experience of living donor liver transplantation (LDLT) to liver resections, the ratio between FLR and body weight (FLR / BW) has been introduced,^[3]^ and is considered to be insufficient when less than 0.5 and 0.8 in patients with healthy and damaged livers, respectively.^[4]^ This formula avoids the problem with nonfunctional tumor mass,^[5]^ and could be useful to establish the indication for associating liver partition and portal vein ligation for staged hepatectomy (ALPPS).

There are 2 ways to increase the FLR / BW ratio; either increase the FLR or decrease the body weight. Here, we present the first case to combine both mechanisms by performing a simultaneous Tourniquet-ALPPS (T-ALPPS) procedure and sleeve gastrectomy to further increase the FLR/BW ratio. This manuscript was prepared following the SCARE guidelines.^[6]^

## 2 Patient presentation

A 54-year-old woman who was morbidly obese (weight: 150 kg, height: 1.6 m, body mass index: 58.59) and had type 2 diabetes mellitus presented to emergency room with abdominal pain and symptoms of intestinal obstruction. The computed tomography (CT) scan showed sigmoid cancer with multiple bilateral liver metastases (one involved the right and middle hepatic vein). She then underwent an emergency sigmoidectomy (pT4N0M1, triple wild type K-RAS, N-RAS and B-RAF), and received ten cycles of FOLFIRI-CETUXIMAB. The liver metastases partially responded, as seen with a CT and positron emission tomography (PET) scan. The FLR was 480 cc, representing 30.3% of the total liver volume, and the FLR/BW ratio was 0.31. The FLR was clearly insufficient and the patient was also candidate for surgical weight loss. After discussing the case in a multidisciplinary committee and obtaining written informed consent by the patient, we decided to perform a T-ALPPS procedure combined with a simultaneous sleeve gastrectomy with interstage chemotherapy.

### 2.1 Surgical technique

**Figure 1:**
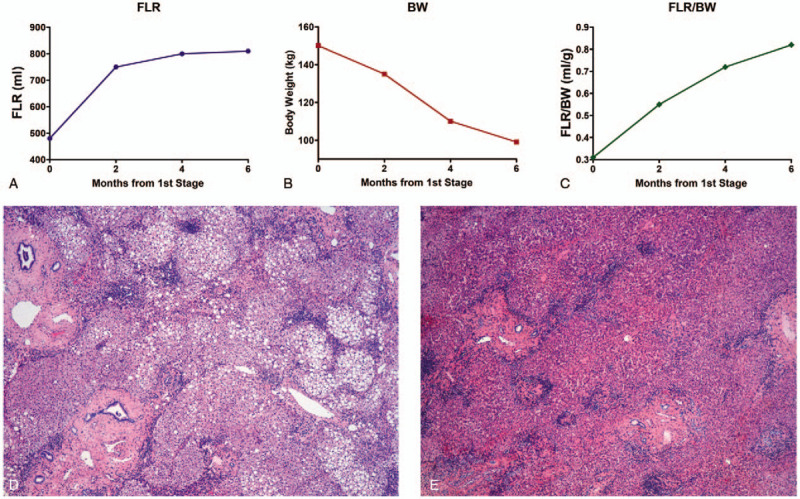
Interstage evolution of FLR (A), body weight (B) and the ratio between the FLR and body weight (C). Macrovesicular steatosis of up to 50% in stage 1 (D, H&E 40×), that decreased to between 1% and 10% in stage 2 (E, H&E 40×).

The T-ALPPS technique was performed as described previously.^[7,8]^ We cleaned the FLR, occluded the portal vein, and placed the tourniquet. Finally, we performed a sleeve gastrectomy. The macrovesicular steatosis in the liver specimen was around 50% (Fig. 1 D).

The post-operative course was uneventful, and the patient was discharged on the 5th post-operative day. Nine cycles of interstage chemotherapy were administered according to the same pre-operative scheme, and there was no disease progression during this time. Six months after the first stage of T-ALPPS, the FLR increased by 68.8% to 810 cc (Fig. 1A), and the patient lost 59.3% of the excess weight (51 kg; Fig. 1B). Further, the FLR / BW ratio was 0.82 (Fig. 1C). Next, the second stage of the T-ALPPS procedure was performed by completing the right trisectionectomy. The liver steatosis in the specimen after the second stage decreased to between 1% and 10% (Fig. 1E). The only post-operative complication was a transient increase of INR treated exclusively with vitamin K. The patient was discharged on the 12th post-operative day. Adjuvant chemotherapy was not administered and actually, four months after the second stage, the patient is alive without recurrence.

## 3 Discussion

To our knowledge, this is the first case in which a sleeve gastrectomy was performed during the first stage of ALPPS in order to reduce the body weight with the purpose of increasing the FLR/BW ratio. The association between liver surgery and sleeve gastrectomy has been described exclusively in the context of liver transplantation to decrease morbi-mortality, especially in patients with a body mass index > 35.^[9]^ Obesity represents a major threat to the development of life-threatening postoperative complications, and may even be a contraindication for surgery, depriving some patients of the only potentially curative treatment. This is especially true for obese patients who require extensive liver resection, since the FLR must be proportional with body weight.

Several methods, such as portal vein embolization and the classic two stage hepatectomy have been developed to operate on patients with an inadequate FLR, however, they obtain an increase in volume lower than 50%. Using the T-ALPPS technique, a variant of the classic ALPPS procedure, we usually obtain a higher increase in volume with a lower morbidity rate. In the present clinical case, we associated this effective hypertrophy technique with a bariatric surgery, obtaining a significant weight loss while the FLR volume grew. These synergic effects facilitated the achievement of an adequate FLR/BW ratio, allowing for safer extended liver resection. In this setting, the classic concept of ALPPS, in which the 2 stages must be carried out within 10 to 15 days, should be modified since it is mandatory to administer interstage chemotherapy waiting to achieve an adequate ratio. In addition, weight loss produced by sleeve gastrectomy could reduce liver steatosis, and could improve glycemic control, both of which are critical factors for obese patients with chemotherapy-related liver damage.

Despite obesity being a recognized risk factor involved in the carcinogenesis of multiple tumors,^[10]^ the role of the bariatric surgery in the oncological setting is not well established. To date, only a few clinical cases have been published.^[11]^ Thus, further studies are necessary to establish whether bariatric surgery should have a role in the context of cancer treatments.

In conclusion, taking into account the limitations of a clinical case report, we believe that performing a sleeve gastrectomy in the first stage of the ALPPS procedure could be feasible and beneficial in select obese patients. A multidisciplinary approach that includes a bariatric team is mandatory for the management of these complex patients.

## Acknowledgments

We would like to thank Editage (www.editage.com) for English language editing.

## Author contributions

XXX.
